# Hydrogen Bond Arrangement Is Shown to Differ in Coexisting Phases of Aqueous Two-Phase Systems

**DOI:** 10.3390/biom11121787

**Published:** 2021-11-30

**Authors:** Pedro P. Madeira, Amber R. Titus, Luisa A. Ferreira, Alexander I. Belgovskiy, Elizabeth K. Mann, Jay Adin Mann, William V. Meyer, Anthony E. Smart, Vladimir N. Uversky, Boris Y. Zaslavsky

**Affiliations:** 1 Department of Chemistry, Centro de Investigacao em Materiais Ceramicos e Compositos, 3810-193 Aveiro, Portugal; p.madeira@ua.pt; 2 Cleveland Diagnostics, 3615 Superior Ave., Cleveland, OH 44114, USA; amber.titus@ClevelandDx.com (A.R.T.); Luisa.Ferreira@cleveland-diagnostics.com (L.A.F.); alexander.belgovskiy@clevelanddx.com (A.I.B.); 3 Department of Physics, Kent State University, Kent, OH 44242, USA; emann@kent.edu; 4 Department of Chemical and Biomolecular Engineering, Case Western Reserve University, Cleveland, OH 44242, USA; j.mann@case.edu; 5 Scattering Solutions, Inc., Cleveland, OH 44242, USA; william.v.meyer@sbcglobal.net; 6 Scattering Solutions, Inc., Costa Mesa, CA 02138, USA; anthonyericsmart@gmail.com; 7 Department of Molecular Medicine, Byrd Alzheimer’s Research Institute, Morsani College of Medicine, University of South Florida, Tampa, FL 33620, USA; vuversky@usf.edu

**Keywords:** aqueous two-phase system, water structure, liquid–liquid phase separation, solvent properties, attenuated total reflection–Fourier transform infrared spectroscopy

## Abstract

Analysis by attenuated total reflection–Fourier transform infrared spectroscopy shows that each coexisting phase in aqueous two-phase systems has a different arrangement of hydrogen bonds. Specific arrangements vary for systems formed by different solutes. The hydrogen bond arrangement is shown to correlate with differences in hydrophobic and electrostatic properties of the different phases of five specific systems, four formed by two polymers and one by a single polymer and salt. The results presented here suggest that the arrangement of hydrogen bonds may be an important factor in phase separation.

## 1. Introduction

Aqueous two-phase systems (ATPSs) are formed in mixtures of two different polymers, such as polyethylene glycol (PEG) and Ficoll, Dextran or polyvinylpyrrolidone (PVP), or a single polymer (e.g., PEG or PVP) and an inorganic salt, such as sodium sulfate or phosphate, in water when their concentrations exceed a certain threshold. The separation of an aqueous mixture of two polymers into two coexisting phases was suggested [1,2,3] to serve as a model of a liquid–liquid phase separation (LLPS). The LLPS mechanism is of fundamental interest because it drives biogenesis of numerous membrane-less organelles (MLOs) abundantly found in prokaryotic and eukaryotic cells [4,5,6,7,8,9,10,11,12,13,14,15,16,17,18]. The chemical nature of compounds responsible for phase separation in simple ATPSs and much more complex MLOs is different. Although synthetic polymers differ from biological macromolecules, such as proteins and nucleic acids, the underlying physical principles of phase separation may well be the same. LLPS is currently classified as associative or segregative [19]. The associative LLPS in aqueous media involves the formation of coacervates, i.e., complexes of oppositely charged polyelectrolytes forming a concentrated phase, which separates from the diluted phase. In systems without coacervates, temperature-dependent associative LLPS can still occur [20,21,22]. Segregative LLPS results in the separation of two (or more) aqueous phases, each enriched in one of the polymers in the initial mixture.

In aqueous solutions, the solvent properties of water are important in both types of LLPS [23,24,25]. Therefore, ATPSs can be used as model systems for the comprehensive analysis of the principles governing LLPS in aqueous media.

Phase separation in ATPSs results in the formation of two immiscible coexisting phases each consisting of water enriched by one or the other polymer or salt. Each of the phases typically contains well over 80% water on a molal basis, suitable for biomacromolecules, such as proteins and nucleic acids [26]. Water structures in the coexisting phases of ATPSs have been suggested to differ [27].

Several recent publications discuss the molecular mechanisms of liquid–liquid phase separation in biological and model systems (e.g., [28,29]) but ignore any effects of the aqueous medium itself. However, many observed effects may be explained by considering that the aqueous medium may itself have complex properties [30,31].

Analysis of the solvent properties of the aqueous media in the phases performed with solvatochromic dyes [32] showed that the solvent properties, such as the solvent dipolarity/polarizability, π*, representing dipole–dipole and dipole-induced dipole interactions, hydrogen bond donor acidity, α, and hydrogen bond acceptor basicity, β, are different and depend upon the composition of the phases. Analysis of the differences between the relative hydrophobicity and electrostatic properties of the phases in different ATPSs performed by studies of the partitioning of the homologous series of dinitrophenylated (DNP-) amino acids with aliphatic alkyl side-chains, such as sodium salts of DNP-glycine, DNP-alanine, DNP-norvaline, DNP-norleucine, and DNP-amino-octanoic acid, demonstrated [33] that the electrostatic and hydrophobic properties of the coexisting phases in ATPSs are also different. The partition behavior of various solutes, including proteins in ATPSs, is governed by the solvent properties of the phases. Phase separation in ATPSs and solute partitioning between coexisting phases may serve as a model for the in vivo processes observed in the formation and functioning of membrane-less organelles.

We have reported recently [34] that the hydrogen bond network of water depends on the specific solute. Attenuated total reflection–Fourier transform infrared (ATR-FTIR) spectroscopy was used [34] to examine the spectra of the OH-stretch band in aqueous solutions of inorganic salts, trimethylamine N-oxide, urea, and the polymers PEG, PVP, and a copolymer of ethylene glycol and propylene glycol (Ucon) at various concentrations. As earlier published [34], the decomposition of the band into four Gaussian components peaking at 3080, 3230, 3400, and 3550 cm^−1^ fits, with minimized residuals, every compound examined. The inspection of the covariance matrices indicated the adequacy of this approach. Our data appear sufficient to show that the simple model of these four components may represent four different subpopulations of water with different H-bond arrangements. Although the experimentally estimated relative contributions of these components depend on the solute type and concentration, the physical dimensions of each subpopulation are currently unknown. The fractional contribution of one or more subpopulations correlates strongly with previously reported experimentally measured solvent features of water such as solvent dipolarity/polarizability, π*, solvent H-bond donor acidity, α, and solvent H-bond acceptor basicity, β [30].

We suggested [34] that water includes an ensemble of exactly four different subpopulations of molecules with various hydrogen bond strengths, geometry, and molecular arrangements depending on the solute. Here, we extend this approach to the coexisting phases of several ATPSs.

The purpose of the present study is to explore whether the arrangements of hydrogen bonds in water differs in the coexisting phases of ATPSs formed by two polymers and by a single polymer and inorganic salt. We also explored whether these differences are correlated with the solvent properties of the phases established previously [33,35] and investigated in this work for two additional ATPSs.

## 2. Materials and Methods

### 2.1. Materials

Polyethylene glycol (PEG-8000, Lot# SLBW6815) with molecular weight (Mw) of 8000 Da, polypropylene glycol (PPG-400, Lot# BCBT9613) with Mw of 400 Da, and polyvinylpyrrolidone (PVP-40,000 Lot# WXBD4555V and Lot# WXBB3898V) with Mw of 40,000 Da were obtained from Sigma-Aldrich (St. Louis, MO, USA). Polyacrylamide (PAM-10,000, Lot# A804225) with Mw of 10,000 Da (50% wt. in water solution) was purchased from Polysciences, Inc. (Warrington, PA, USA). Ucon 50-HB-5100 (Ucon-3930, Lot# SJ1955S3D2), a random copolymer of 50% ethylene oxide and 50% propylene oxide, with Mw of 3930, was purchased from Dow-Chemical (Midland, MI, USA). Sodium sulfate, sodium phosphate monobasic, sodium phosphate dibasic, and KCl of analytical reagent grade were purchased from Fisher Scientific (Waltham, MA, USA) and used without further purification. Ultrapure water purified using a Millipore Milli-Q lab water system was used for preparation of all solutions.

Dinitrophenylated (DNP-) amino acids (DNP-alanine, DNP-norvaline, DNP-norleucine, and DNP-α-amino-N-octanoic acid) were purchased from Sigma-Aldrich. The sodium salts of the DNP-amino acids were prepared by titration.

### 2.2. Methods

#### 2.2.1. ATR-FTIR Measurements and Spectra Analysis

Details of ATR-FTIR measurements as described in [34]. ATR-FTIR spectra for each sample were measured in two separately prepared solutions and two aliquots from each phase from each ATPS using a Spectrum Bx FT-IR spectrometer (Perkin-Elmer, Boston, MA, USA) equipped with single reflection Golden Gate diamond ATR (Specac, London, UK). All measurements were performed at about 25 °C using 20 scans for each sample and 24 scans for background in the spectral range of 4000–1000 cm^−1^ with resolution of 2 cm^−1^. The spectra were reproducible to within ±1 cm^−1^.

#### 2.2.2. Analysis of Spectra

ATR-FTIR spectra were analyzed with custom software written in Wolfram Mathematica (version 9). The software performed peak analysis by fitting the data using ‘NonlinearModelFit’ function, the model function being a sum of two, three, four, and five Gaussians with floated central frequencies. We found that the best and most reliable fits are obtained with four Gaussians with peak locations [34] (3080, 3230, 3400, and 3550 cm^−1^). The program displays the results graphically (raw data, model function fit, and individual Gaussians) and reports the calculated parameter values for each individual peak with metrics of each fit quality.

#### 2.2.3. Preparation of Aqueous Two-Phase Systems

Aqueous two-phase systems were prepared as described in [23,26,32,33]. The ATPSs of the compositions listed in Table S1 were prepared separately for FTIR spectra measurements and partitioning of DNP-amino acids Na salts.

Stock solutions of 50% wt. PEG-8000, 40% wt. Ucon-3930, 32.1% wt. PVP-40,000, 50% wt. PAM-10,000, and 20.3% wt. Na_2_SO_4_ were prepared in water. Sodium phosphate buffer (NaPB, pH 7.4) stock solution (0.5 M) was prepared by mixing 3.28 g monobasic sodium phosphate and 27.15 g disodium hydrogen phosphate heptahydrate in 100 mL of water.

For the FTIR measurements, 1.2 g ATPS was prepared by weight in a 1.5 mL tube. The prepared systems were vigorously mixed with vortex mixing and centrifuged at 4500 g for 30 min using Hettich Universal 320R centrifuge system.

Samples from both phases were collected for FTIR analysis (12 g ATPS system prepared by weight). A pipette was used to remove the top phase, while the bottom phase was removed through the drain of the separatory funnel.

For partitioning experiments, the ATPSs of total weight 0.5 g (after addition of the solute sample, see below) were prepared by dispensing appropriate amounts of the above stock solutions into a 1.2 mL microtube using a Hamilton (Reno, NV, USA) ML-400 four-probe liquid-handling workstation.

#### 2.2.4. Partitioning Experiments

An automated instrument for performing aqueous two-phase partitioning, the Automated Signature Workstation, ASW (Analiza, Inc., Cleveland, OH, USA), was used for the partitioning experiments. The ASW system is based on the ML-4000 liquid-handling workstation (Hamilton Company, Reno, NV, USA) integrated with a UV-VIS microplate spectrophotometer (FluoStar Omega (BMG Labtech, Cary, NC, USA)). Solutions of DNP-amino acids Na salts were prepared in water at concentrations of 10 mM. Varied amounts (e.g., 0, 5, 10, 15, 20, and 25 μL) of compound solution and the corresponding amounts (e.g., 75, 60, 45, 30, 15, and 0 μL) of water were added to a set of the same polymers/buffer/salt mixtures. The systems were then vortexed and centrifuged (Hettich, Universal 320R centrifuge) for 30–60 min at 3500× *g* at 23 °C to accelerate phase settling. The top phase in each system was removed, the interface discarded, and aliquots from the top and bottom phases were withdrawn in duplicate for analysis. For the analysis of the compounds partitioning, aliquots of 80 μL from both phases were diluted with water up to 800 μL in 1.2 mL microtubes. Following vortexing and a short centrifugation (12 min), aliquots of 250 μL were transferred into microplate wells, and the UV-VIS plate reader was used to measure optical absorbance at wavelengths previously determined to correspond to maximum absorption (362 nm). In all measurements, the dilution factors used for the upper and lower phases was taken into account, and correspondingly diluted pure phases were used as blank solutions. The partition coefficient, K, is defined as the ratio of the solute concentration in the top phase to that in the bottom phase. The K-value for each solute was determined as the slope of the concentration (absorbance) in the top phase plotted as a function of the concentration in the bottom phase averaged over the results obtained from two to four partition experiments carried out at the specified composition of the system. The deviation from the average K value was always less than 3%.

#### 2.2.5. Analysis of Electrostatic and Hydrophobic Properties of the Phases

The difference between the electrostatic and hydrophobic properties of the coexisting phases was determined for each ATPS by partitioning a homologous series of sodium salts of dinitrophenylated (DNP-) amino acids with the aliphatic alkyl side-chains of increasing length, alanine, norvaline, norleucine, and α-amino-n-octanoic acid as described previously [26,32,33,35]. Partition coefficients of these compounds are presented graphically below (see Section 3), where the logarithms of their partition coefficients are plotted against the length of the side chain expressed in equivalent number of methylene groups, N_c_. The N_c_ values for the DNP-amino acids used are DNP-alanine Na—1.31, DNP-norvaline Na—2.65, DNP-norleucine Na—3.75, and DNP-α-amino-n-octanoic acid Na—6.30.

## 3. Results

**Figure 1:**
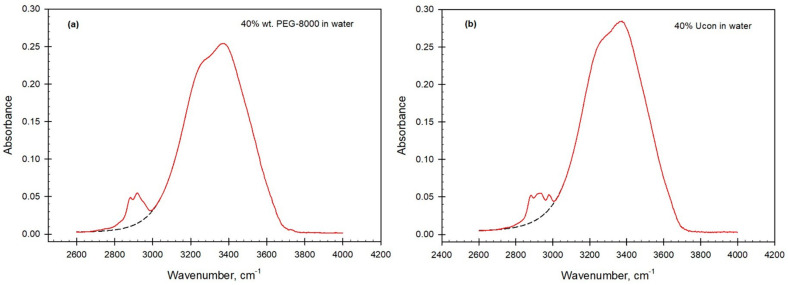
ATR−FTIR spectra of the OH-stretch band observed (solid red) and interpolated (dashed black) in 40% wt. aqueous solutions of (**a**) PEG-8000 and (**b**) Ucon-3230.

We used ATR-FTIR spectroscopy to examine the OH-stretch bands in coexisting phases of five ATPSs formed by different pairs of polymers (PEG-8000-Ucon-3230 at two ionic compositions, PEG-8000-PVP-40,000, PEG-8000-polyacrylamide (PAM-10,000) and by polymer and salt (PEG-8000-Na_2_SO_4_). Figure 1 shows the typical OH-stretch band in solution of PEG-8000 and Ucon-3230 in ultrapurified water. A few relatively small peaks in the spectrum are observed in the 2750–2980 cm^−1^ region and are likely to originate from C-H stretch—their intensity increases with the polymer concentration in solution. Similar peaks are also observed in solutions of Ucon. Since these peaks may influence the decomposition of the OH-stretch band into four Gaussian curves, we removed these data points and interpolated the peak absorption values in this interval as shown in Figure 1 and explained in the Supplementary Material, Section S1. Although this interpolation introduces only insignificant uncertainty in the estimated values of relative contributions of Gaussian components, some uncertainty may remain because Ucon and PEG each contain a single hydroxyl group per molecule, which absorbs in the same region as pure water under similar conditions of temperature and pressure.

**Figure 2:**
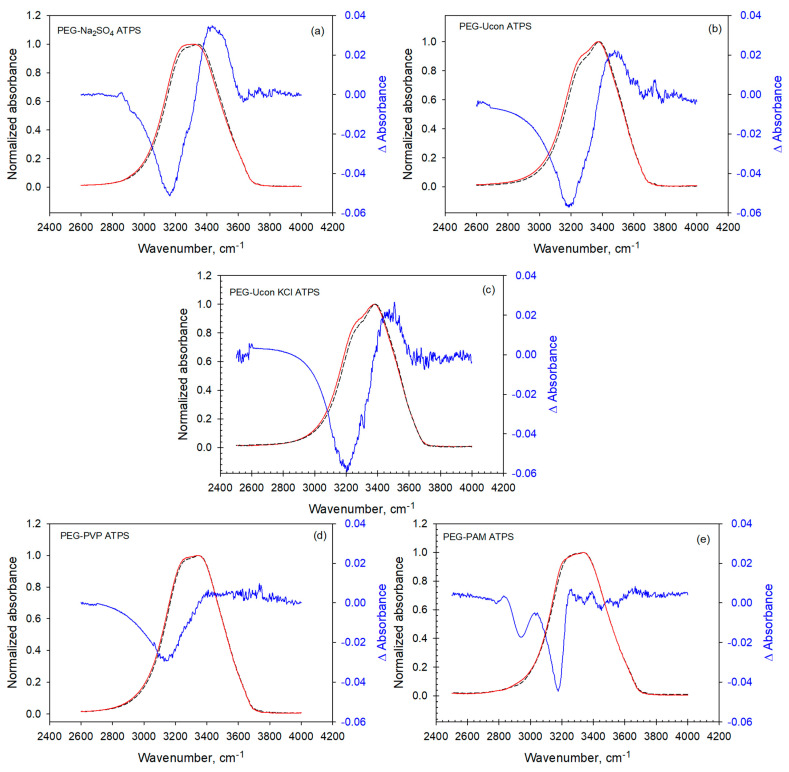
(**a**–**e**) ATR-FTIR spectra of the OH-stretch bands of water in the coexisting phases of ATPSs show the differences between the spectra. Top phase—dashed black line; bottom phase—solid red line; difference (right ordinate) between the spectra—solid blue line, calculated as the difference between the spectrum of the top phase minus the spectrum of the bottom phase.

The spectra of the coexisting phases in the region of 4000–2500 cm^−1^ are presented in Figure 2a–e. The spectra of the two phases in PEG-Na_2_SO_4_ (a) and PEG-Ucon ATPSs (b) and (c) show a significant difference (blue, right ordinate) in absorbance between 3300 and 3600 cm^−1^, whereas for PEG-PVP (d) and PEG-PAM (e) in this region the difference becomes almost imperceptible above 3300 cm^−1^. Furthermore, the absolute differences are almost a factor of two greater for the first three ATPSs (a), (b), and (c) than for last two (d) and (e).

The OH-stretch band is typically made up of several components, each assigned to water molecules existing in a different H-bond environment [30], where a satisfactory fit was always obtained with exactly four components, in agreement with the data obtained by Kitadai et al. [36]. These four different peaks may be associated with collections of water molecules with: (I) 3080 cm^−1^—four tetrahedrally arranged hydrogen bonds, (II) 3230 cm^−1^—four distorted hydrogen bonds, (III) 3400 cm^−1^—four or three loosely arranged hydrogen bonds, and (IV) 3550 cm^−1^—three, two or single hydrogen bonds.

**Figure 3:**
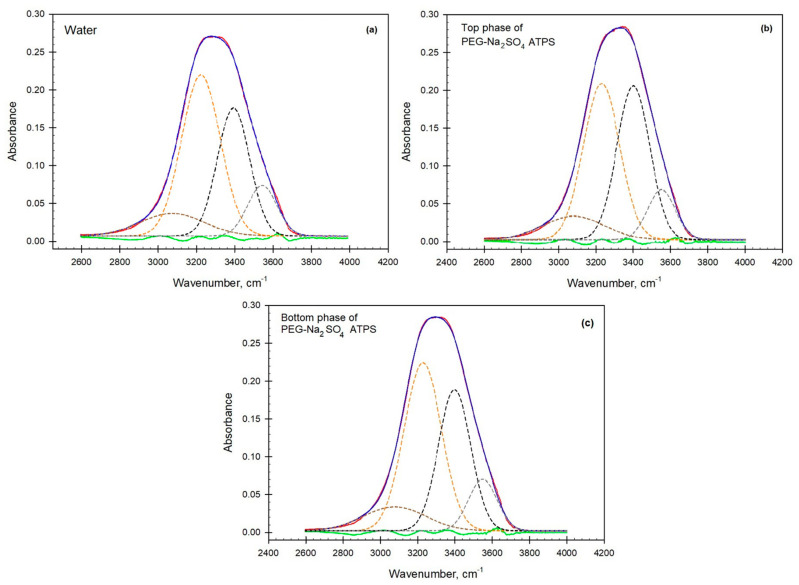
(**a**–**c**). Decomposition of the ATR-FTIR spectra of OH-stretch band in (**a**) ultrapurified water, (**b**) top, and (**c**) bottom phases of PEG-Na_2_SO_4_ ATPS.

Based on our internally consistent empirical measurements, this assignment is a useful approximation to complex hydrogen-bond networks in water [37]. We conjectured [30] that these collections of differently ordered water are distributed throughout bulk water with ratios that depend on the type and concentration of solute (see Figure 3a). We do not, however, speculate upon the scale and/or nature of the physical distributions, whether locally clustered, intercalated, or dendritic. The decomposition of the spectral band into four Gaussian components is shown in Figure 3a–c.

**Figure 4:**
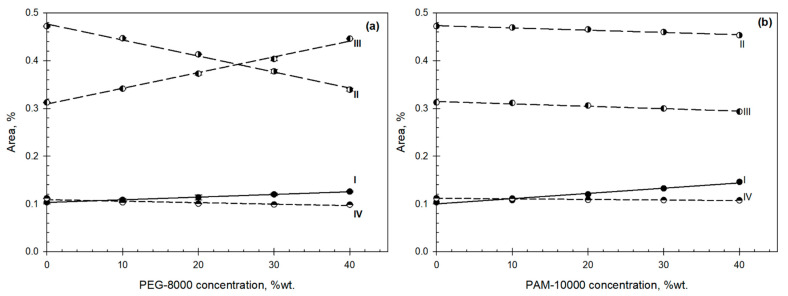
(**a**,**b**). Contribution of each Gaussian component of water into overall ATR-FTIR spectra of OH-stretch band for the PEG-8000 (**a**) and PAM-10000 (**b**) as a function of the polymer concentration. (I (3080 cm^−1^), II (3230 cm^−1^), III (3400 cm^−1^), and IV (3550 cm^−1^)).

Figure 4a,b shows the estimated relative percentage area under each fitted Gaussian curve for each component as the function of the solute concentration with raw data in the Supplementary Material, Table S2.

The estimates of the relative percentage area for each Gaussian component for water in the phases of the ATPSs are presented in the Supplementary Material, Table S3.

**Figure 5:**
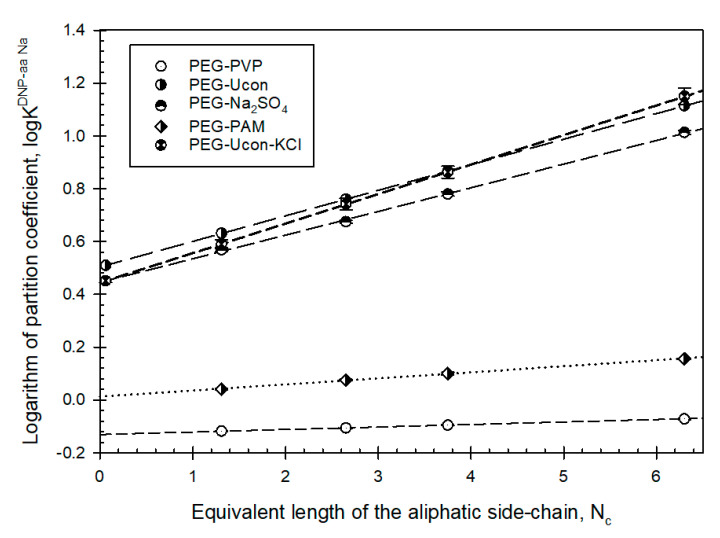
Logarithm of the partition coefficient, log K^DNP-aa Na^, value for sodium salts of DNP—amino acids with aliphatic side chains in aqueous two-phase systems indicated as a function of equivalent length of the side chain, N_C_, expressed in terms of the equivalent number of CH_2_ units.

Figure 5 shows the dependence of the logarithm of the partition coefficients of the DNP-amino acids Na-salts in PEG-PVP and PEG-PAM ATPSs on the length of the side chain expressed in an equivalent number of methylene groups, N_c_, and also shows that the data in each ATPS are as described [33] by:

log_10_K_i_^DNP-aa^ = c_i_ + E_i_*N_c_ (1)

where K^i^_DNP-AA_ is the partition coefficient of a DNP-amino acids Na-salt; N_c_ is the equivalent number of CH_2_ groups in the side chain; and E and c are constants for the given ith ATPS characterizing the difference between the relative hydrophobicity and electrostatic properties of the phases correspondingly. Table 1 lists E_i_ and c_i_ values for the ATPS under study.

Data supporting Figure 5 are given in the Supplementary Material, Table S4.

## 4. Discussion

**Figure 6:**
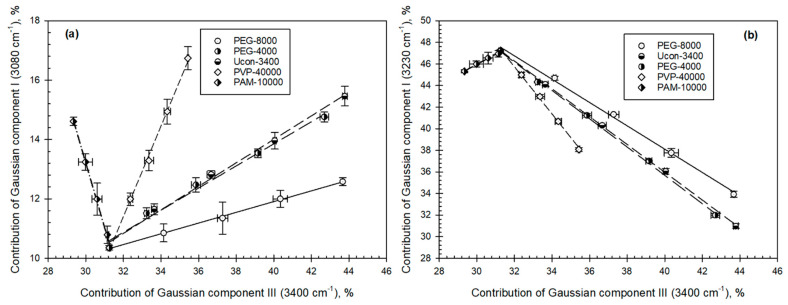
(**a**,**b**). Relationships between (**a**) contributions of Gaussian component I (I^3080 cm^−1^^) and III (I^3400 cm^−1^^) and (**b**) contributions of Gaussian component II (I^3230 cm^−1^^) and III (I^3400 cm^−1^^) in aqueous solutions of individual polymers indicated over the concentration range of 0–40% wt.

Analysis of the four Gaussian components of the OH-stretch band estimated for the solutions of individual polymers shows (Figure 6) the existence of two sets of linear relationships described by:

I_j_^3080^ = k_0j_ + k_1j_I_j_^3400^   I_j_^3230^ = k_0j_ + k_1j_I_j_^3400^ (2)

where I_j_^3080^, I_j_^3230^, and I_j_^3400^ are contributions of the Gaussian components I (3080 cm^−1^), II (3230 cm^−1^), and III (3400 cm^−1^), respectively—component IV (3550 cm^−1^) changes only insignificantly for all compounds studied; k_0_ and k_j_ are coefficients; and the subscript j denotes the polymer examined.

Various solvent features of aqueous solutions of different individual compounds (except some inorganic salts) may be described by the contributions of Gaussian curves III and/or IV [34]. Because these Gaussian components represent the least ice-like water structures, we conjecture that they are probably responsible for the solvent properties of aqueous solutions and phases of ATPSs. We examined the differences between hydrophobic and electrostatic properties of coexisting phases of the ATPSs under study, correlating them with those between the contributions of Gaussian curve III for the same ATPSs.

**Figure 7:**
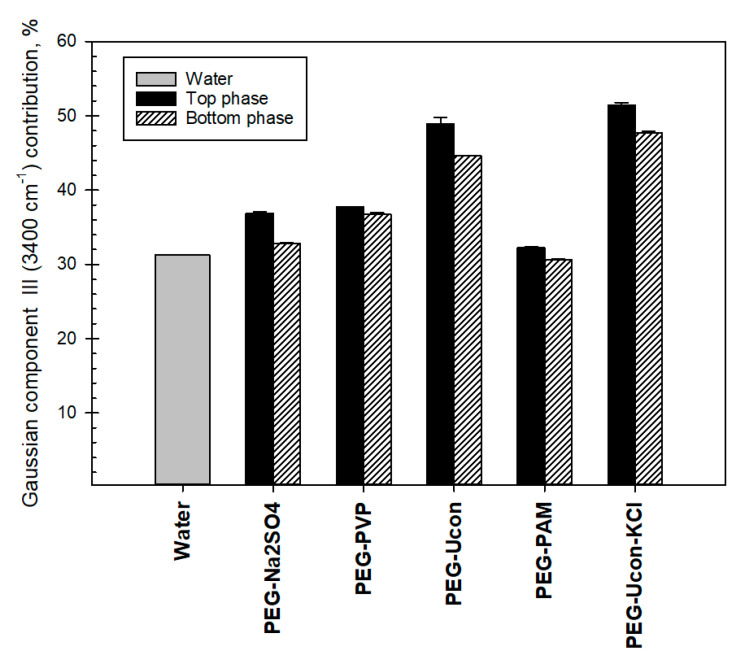
Differences between relative contributions (%) of Gaussian component III in the OH-stretch band of water in the phases (I^3400 cm^−1^^) of the aqueous two-phase systems indicated and contribution of the component III in pure water.

Figure 7 shows the differences between pure water and contributions of Gaussian component III in the FTIR OH-stretch band in the two phases of the ATPS.

**Figure 8:**
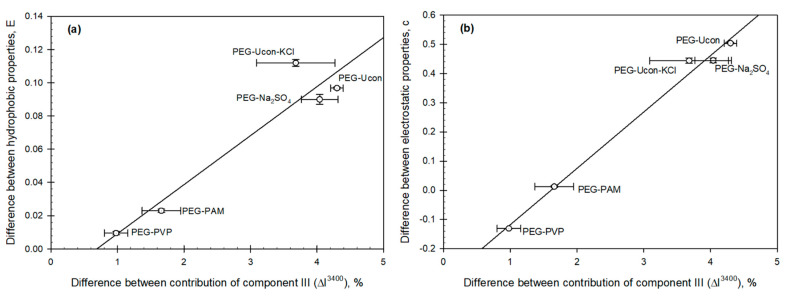
Difference between (**a**) hydrophobic properties (parameter E) of the phases and (**b**) electrostatic properties (parameter c) as a function of the difference between contributions of Gaussian component III in the OH—stretch band of water in the coexisting phases (ΔI^3400 cm^−1^^) of the aqueous two-phase systems indicated (parameters E and c as defined in Equation (1)).

Analysis of the differences between the hydrophobic and electrostatic properties of the coexisting phases (parameters E and c in Equation (1), accordingly) shows that both these parameters are linearly related to those between the contributions of Gaussian component III (I^3400 cm^−1^^) in the OH-stretch band in the phases of all the ATPSs examined as illustrated graphically in Figure 8a,b.

Parameter c representing the difference between the electrostatic properties of the coexisting phases is generally the most sensitive to the ionic composition of an ATPS, and therefore, its linear relationship with the ΔI^3400 cm^−1^^ parameter for PEG-Ucon-KCl and especially PEG-sulfate ATPS is intriguing. More general conclusions, while supported by the limited number of ATPSs examined, require further evidence.

This study is the first to find the differences between the arrangement of H-bonds in the coexisting phases of ATPSs and the correlations reported. This observation raises important questions: (i) How do the ionic and nonionic additives affect the arrangement of H-bonds? (ii) How do the molecular weight and/or size of phase-forming polymers influence the arrangement of H-bonds? (iii) At what polymer concentration does the arrangement of H-bonds initiate phase separation? Answers to these questions are currently of interest in our laboratories.

## 5. Conclusions

Analysis of the OH-stretch band of water with ATR-FTIR spectroscopy shows that a model based on exactly four Gaussian components positioned at 3080, 3230, 3400, and 3550 cm^−1^ is both necessary and sufficient to describe the properties of aqueous media in coexisting phases of ATPSs formed by different combinations of nonionic polymers and a single polymer and inorganic salt.

An additional justification for using the chosen positions for each of the four peaks is that free-fitted peak positions for pure water and for the polymer solution with the polymer spectrum deweighted are essentially identical, which is not the case for five Gaussian fits.

Although our bulk transmission measurements indicate the relative amount of each subpopulation of water with different H-bond arrangements, assuming that a representative ensemble is included in each line of sight, all we can say about the size of the subpopulation is that they must be much smaller than the size of the specimen measuring volume, on the order of microliters. Using the current analyses of these measurements, we make no predictions about the locations, sizes, and shapes of the four types of subpopulations, even though their relative contributions seem well determined.

The consistency and shape of the extremely small residuals may indicate that the distributions that we are actually fitting with symmetric Gaussians may have a trace of skewness. This is approximately supported by noting that the peaks of the residuals tend to cluster near the steepest slopes of each Gaussian, implying that any added skewness might further reduce these already small residuals.

According to this model, the component at 3400 cm^−1^ represents the subpopulation/cluster of water with H-bond arrangements from water molecules with three and two hydrogen bonds. The contribution of this component correlates strongly and linearly with the solvent properties of the phases in different ATPSs. Hence, the electrostatic and hydrophobic properties are strongly correlated with the arrangement of hydrogen bonds in the coexisting phases.

## Figures and Tables

**Table 1:** Solvent properties of studied aqueous two-phase systems.

ATPS	E	c	ΔI^3400 cm^−1^^
PEG-Ucon ^a^	0.0968 ± 0.0006	0.504 ± 0.002	0.0430 ± 0.0094
PEG-Ucon-KCl ^a^	0.112 ± 0.002	0.444 ± 0.009	0.0368 ± 0.0059
PEG-PVP	0.0095 ± 0.001	−0.131 ± 0.001	0.0098 ± 0.0018
PEG-PAM	0.023 ± 0.001	0.013 ± 0.003	0.0166 ± 0.0029
PEG-Na_2_SO_4_ ^b^	0.090 ± 0.003	0.445 ± 0.008	0.0404 ± 0.0028

^a^ Data from [33]. ^b^ Data from [35].

## Data Availability

The data presented in this study are available within the article and supplementary material.

## Supplementary Materials

The following are available online at https://www.mdpi.com/article/10.3390/biom11121787/s1, Table S1: Compositions of ATPSs examined (NaPB—sodium phosphate buffer, pH 7.4), Table S2: Contributions of Gaussian components (I–IV) into spectra of the OH-stretch band in aqueous solutions of individual polymers, Table S3: Relative contributions of Gaussian components I–IV in the FTIR spectra of the coexisting phases of ATPSs indicated, Table S4: Partition coefficients, K, of sodium salts of DNP-amino acids in ATPS indicated.

## References

[1] Titus A.R., Ferreira L.A., Belgovskiy A.I., Kooijman E.E., Mann E.K., Mann J.A., Meyer W.V., Smart A.E., Uversky V.N., Zaslavsky B.Y. (2020). Interfacial tension and mechanism of liquid-liquid phase separation in aqueous media. Phys. Chem. Chem. Phys..

[2] Sakuta H., Fujimoto T., Yamana Y., Hoda Y., Tsumoto K., Yoshikawa K. (2019). Aqueous/Aqueous Micro Phase Separation: Construction of an Artificial Model of Cellular Assembly. Front. Chem..

[3] Aumiller W.M., Keating C.D. (2017). Experimental models for dynamic compartmentalization of biomolecules in liquid organelles: Reversible formation and partitioning in aqueous biphasic systems. Adv. Colloid Interface Sci..

[4] Nesterov S.V., Ilyinsky N.S., Uversky V.N. (2021). Liquid-liquid phase separation as a common organizing principle of intracellular space and biomembranes providing dynamic adaptive responses. Biochim. Biophys. Acta Mol. Cell Res..

[5] Azaldegui C.A., Vecchiarelli A.G., Biteen J.S. (2021). The emergence of phase separation as an organizing principle in bacteria. Biophys. J..

[6] Jalihal A.P., Schmidt A., Gao G., Little S.R., Pitchiaya S., Walter N.G. (2021). Hyperosmotic phase separation: Condensates beyond inclusions, granules and organelles. J. Biol. Chem..

[7] Hondele M., Heinrich S., De Los Rios P., Weis K. (2020). Membraneless organelles: Phasing out of equilibrium. Emerg. Top. Life Sci..

[8] Zhang H., Ji X., Li P., Liu C., Lou J., Wang Z., Wen W., Xiao Y., Zhang M., Zhu X. (2020). Liquid-liquid phase separation in biology: Mechanisms, physiological functions and human diseases. Sci. China Life Sci..

[9] Uversky V.N., Finkelstein A.V. (2019). Life in Phases: Intra- and Inter- Molecular Phase Transitions in Protein Solutions. Biomolecules.

[10] Bracha D., Walls M.T., Brangwynne C.P. (2019). Probing and engineering liquid-phase organelles. Nat. Biotechnol..

[11] Feng Z., Chen X., Wu X., Zhang M. (2019). Formation of biological condensates via phase separation: Characteristics, analytical methods, and physiological implications. J. Biol. Chem..

[12] Darling A.L., Zaslavsky B.Y., Uversky V.N. (2019). Intrinsic Disorder-Based Emergence in Cellular Biology: Physiological and Pathological Liquid-Liquid Phase Transitions in Cells. Polymers.

[13] Turoverov K.K., Kuznetsova I.M., Fonin A.V., Darling A.L., Zaslavsky B.Y., Uversky V.N. (2019). Stochasticity of Biological Soft Matter: Emerging Concepts in Intrinsically Disordered Proteins and Biological Phase Separation. Trends BioChem. Sci..

[14] Bentley E.P., Frey B.B., Deniz A.A. (2019). Physical Chemistry of Cellular Liquid-Phase Separation. Chemistry.

[15] Sawyer I.A., Sturgill D., Dundr M. (2019). Membraneless nuclear organelles and the search for phases within phases. Wiley Interdiscip. Rev. RNA.

[16] Zaslavsky B.Y., Ferreira L.A., Darling A.L., Uversky V.N. (2018). The solvent side of proteinaceous membrane-less organelles in light of aqueous two-phase systems. Int. J. Biol. Macromol..

[17] Uversky V.N. (2017). Intrinsically disordered proteins in overcrowded milieu: Membrane-less organelles, phase separation, and intrinsic disorder. Curr. Opin. Struct. Biol..

[18] Uversky V.N. (2017). Protein intrinsic disorder-based liquid-liquid phase transitions in biological systems: Complex coacervates and membrane-less organelles. Adv. Colloid Interface Sci..

[19] Martin N. (2019). Dynamic Synthetic Cells Based on Liquid-Liquid Phase Separation. ChembioChem.

[20] Johansson H.O., Persson J., Tjerneld F. (1999). Thermoseparating water/polymer system: A novel one-polymer aqueous two-phase system for protein purification. Biotechnol. Bioeng..

[21] Ge X., Conley A.J., Brandle J.E., Truant R., Filipe C.D. (2009). In vivo formation of protein based aqueous microcompartments. J. Am. Chem. Soc..

[22] Urry D.W. (1997). Physical chemistry of biological free energy transduction as demonstrated by elastic protein-based polymers. J. Phys. Chem. B.

[23] Ferreira L.A., Cole J.T., Reichardt C., Holland N.B., Uversky V.N., Zaslavsky B.Y. (2015). Solvent Properties of Water in Aqueous Solutions of Elastin-Like Polypeptide. Int. J. Mol. Sci..

[24] Li N.K., Garcia Quiroz F., Hall C.K., Chilkoti A., Yingling Y.G. (2014). Molecular description of the LCST behavior of an elastin-like polypeptide. Biomacromolecules.

[25] Li L., Rumyantsev A.M., Srivastava S., Meng S., de Pablo J.J., Tirrell M.V. (2021). Effect of solvent quality on the phase behavior of polyelectrolyte complexes. Macromolecules.

[26] Zaslavsky B.Y. (1994). Aqueous Two-Phase Partitioning: Physical Chemistry and Bioanalytical Applications.

[27] Zaslavsky B., Bagirov T., Borovskaya A., Gulaeva N., Miheeva L., Mahmudov A., Rodnikova M. (1989). Structure of water as a key factor of phase separation in aqueous mixtures of two non-ionic polymers. Polymer.

[28] Edun D.N., Flanagan M.R., Serrano A.L. (2020). Does liquid-liquid phase separation drive peptide folding?. Chem. Sci..

[29] Hyman A.A., Weber C.A., Julicher F. (2014). Liquid-liquid phase separation in biology. Annu. Rev. Cell Dev. Biol..

[30] Ferreira L.A., Madeira P.P., Breydo L., Reichardt C., Uversky V.N., Zaslavsky B.Y. (2016). Role of solvent properties of aqueous media in macromolecular crowding effects. J. Biomol. Struct. Dyn..

[31] Ferreira L.A., Uversky V.N., Zaslavsky B.Y. (2017). Role of solvent properties of water in crowding effects induced by macromolecular agents and osmolytes. Mol. Biosyst..

[32] Madeira P.P., Reis C.A., Rodrigues A.E., Mikheeva L.M., Zaslavsky B.Y. (2010). Solvent properties governing solute partitioning in polymer/polymer aqueous two-phase systems: Nonionic compounds. J. Phys. Chem. B.

[33] Madeira P.P., Bessa A., Alvares-Ribeiro L., Aires-Barros M.R., Reis C.A., Rodrigues A.E., Zaslavsky B.Y. (2012). Salt effects on solvent features of coexisting phases in aqueous polymer/polymer two-phase systems. J. Chromatogr. A.

[34] da Silva N.R., Ferreira L.A., Belgovskiy A.I., Madeira P.P., Teixeira J.A., Mann E.K., Mann J.A., Meyer W.V., Smart A.E., Chernyak V.Y. (2021). Effects of different solutes on the physical chemical properties of aqueous solutions via rearrangement of hydrogen bonds in water. J. Mol. Liq..

[35] Ferreira L.A., Parpot P., Teixeira J.A., Mikheeva L.M., Zaslavsky B.Y. (2012). Effect of NaCl additive on properties of aqueous PEG-sodium sulfate two-phase system. J. Chromatogr. A.

[36] Kitadai N., Sawai T., Tonoue R., Nakashima S., Katsura M., Fukushi K. (2014). Effects of Ions on the OH Stretching Band of Water as Revealed by ATR-IR Spectroscopy. J. Solut. Chem..

[37] Brini E., Fennell C.J., Fernandez-Serra M., Hribar-Lee B., Luksic M., Dill K.A. (2017). How Water’s Properties Are Encoded in Its Molecular Structure and Energies. Chem. Rev..

