# Supplementary material for: Hydrogen Bond Arrangement Is Shown to Differ in Coexisting Phases of Aqueous Two-Phase Systems
Source: Biomolecules. 2021 Nov 30;11(12):1787. doi: 10.3390/biom11121787 (PMC8698934; doi:10.3390/biom11121787)
Supplement: Supplementary file 1 [file biomolecules-11-01787-s001.zip › biomolecules-1458349-supplementary.pdf]

Supporting Information for the Manuscript

Hydrogen Bond Arrangement is Shown to Differ in Coexisting Phases of Aqueous Two-Phase Systems

Pedro P. Madeira,[a] Amber R. Titus,[b] Luisa A. Ferreira,[b] Alexander I. Belgovsky,[b] Elizabeth K. Mann,[c] J. Adin Mann Jr.,[d] William V. Meyer,[e] Anthony E. Smart,[f] Vladimir N. Uversky,[g] and Boris Y. Zaslavsky[b]

[a] Centro de Investigacao em Materiais Ceramicos e Compositos, Department of Chemistry, Aveiro, Portugal;

[b]Cleveland Diagnostics, 3615 Superior Ave., Cleveland, OH 44114, USA;
[c]Department of Physics, Kent State University, Kent, OH, USA;
[d]Department of Chemical and Biomolecular Engineering, Case Western Reserve University, Cleveland, OH, USA;
[e]Scattering Solutions, Inc., Cleveland, OH, USA;
[f]Scattering Solutions, Inc., Costa Mesa, Ca, USA;
[g]Department of Molecular Medicine and Byrd Alzheimer's Research Institure, Morsani College of Medicine, University of South Florida, Tampa, FL, USA.
*Corresponding Author, E-mail: Boris.Zaslavsky@Cleveland-Diagnostics.com, Phone: +1-216-432-2700.

CONTENT:

Tables S1 to S4

Table S1. Compositions of ATPSs examined (NaPB – sodium phosphate buffer, pH 7.4).

Polymer 1		Polymer 2 (Salt)		Salt/buffer
PEG-8000	16.0 %wt.	PVP-40,000	18.0 %wt.	0.01 M NaPB
PEG-8000	8.0 %wt.	PAM-10,000	16.0 %wt.	0.01 M NaPB
PEG-8000	15.0 %wt.	Ucon-3930	30.0 %wt.	0.01 M NaPB
PEG-8000	15.0 %wt.	Ucon-3930	30.0 % wt.	0.15 M KCl + 0.01 M NaPB
PEG-8000	11.1 %wt.	Na_2SO_4	6.33 %wt.	0.01 M NaPB

Table S2. Contributions of Gaussian components (I-IV) into spectra of the OH-stretch band in aqueous solutions of individual polymers.

Polymer	[Polymer], %wt.	I^{3080}	I^{3230}	I^{3400}	I^{3550}
	-	$0.1035_{0.0001}$	$0.4722_{0.0007}$	$0.3125_{0.0008}$	$0.1118_{0.0003}$
PEG-8000	10.0	$0.1085_{0.0030}$	$0.4469_{0.0018}$	$0.3414_{0.0007}$	$0.1032_{0.0005}$
	20.0	$0.1135_{0.0054}$	$0.4132_{0.0002}$	$0.3728_{0.0027}$	$0.1005_{0.0005}$
	30.0	$0.1200_{0.0028}$	$0.3777_{0.0041}$	$0.4035_{0.0037}$	$0.0988_{0.0004}$
	40.0	$0.1258_{0.0014}$	$0.3392_{0.0029}$	$0.4460_{0.0002}$	$0.0984_{0.0006}$
PAM	10.0	$0.1079_{0.0032}$	$0.4694_{0.0029}$	$0.3115_{0.0045}$	$0.1112_{0.0007}$
	20.0	$0.1199_{0.0017}$	$0.4655_{0.0054}$	$0.3060_{0.0031}$	$0.1085_{0.0011}$
	30.0	$0.1324_{0.0034}$	$0.4599_{0.0028}$	$0.2998_{0.0009}$	$0.1079_{0.0021}$
	40.0	$0.1461_{0.0033}$	$0.4532_{0.0014}$	$0.2934_{0.0012}$	$0.1073_{0.0005}$

Table S3. Relative contributions of Gaussian components I-IV in the FTIR spectra of the coexisting phases of ATPSs indicated

**ATPS**	**Phase**	$\mathbf{I^{3080}}$	$\mathbf{I^{3230}}$	$\mathbf{I^{3400}}$	$\mathbf{I^{3550}}$
PEG-PVP	Upper	$0.1330_{\pm 0.0001}$	$0.3925_{\pm 0.0002}$	$0.3774_{\pm 0.0001}$	$0.0971_{\pm 0.0003}$
	Lower	$0.1422_{\pm 0.0013}$	$0.3945_{\pm 0.0005}$	$0.3676_{\pm 0.0016}$	$0.0958_{\pm 0.0002}$
	$\Delta^{upper-lower}$	$-0.0092_{\pm 0.0015}$	$-0.0020_{\pm 0.0005}$	$0.0098_{\pm 0.0018}$	$0.0013_{\pm 0.0005}$
PEG-PAM	Upper	$0.1024_{\pm 0.0005}$	$0.4712_{\pm 0.0014}$	$0.3226_{\pm 0.0011}$	$0.1040_{\pm 0.0002}$
	Lower	$0.1178_{\pm 0.0045}$	$0.4658_{\pm 0.0047}$	$0.3060_{\pm 0.0018}$	$0.1105_{\pm 0.0016}$
	$\Delta^{upper-lower}$	$-0.0015_{\pm 0.0050}$	$0.0054_{\pm 0.0061}$	$0.0166_{\pm 0.0029}$	$-0.0065_{\pm 0.0018}$
PEG-Ucon	Upper	$0.1611_{\pm 0.0016}$	$0.2485_{\pm 0.0149}$	$0.4891_{\pm 0.0090}$	$0.1014_{\pm 0.0044}$
	Lower	$0.1541_{\pm 0.0008}$	$0.3016_{\pm 0.0009}$	$0.4461_{\pm 0.0004}$	$0.00982_{\pm 0.0013}$
	$\Delta^{upper-lower}$	$0.0070_{\pm 0.0024}$	$-0.0531_{\pm 0.0158}$	$0.0430_{\pm 0.0094}$	$0.0032_{\pm 0.0057}$
PEG-Na_2SO_4	Upper	$0.1193_{\pm 0.0012}$	$0.4094_{\pm 0.0011}$	$0.3691_{\pm 0.0018}$	$0.1023_{\pm 0.0005}$
	Lower	$0.1084_{\pm 0.0008}$	$0.4569_{\pm 0.0004}$	$0.3287_{\pm 0.0009}$	$0.1061_{\pm 0.0005}$
	$\Delta^{upper-lower}$	$0.0109_{\pm 0.0020}$	$-0.0475_{\pm 0.0015}$	$0.0404_{\pm 0.0027}$	$-0.0038_{\pm 0.0010}$
PEG-Ucon-KCl	Upper	$0.1128_{\pm 0.0010}$	$0.2656_{\pm 0.0047}$	$0.5142_{\pm 0.0039}$	$0.1075_{\pm 0.0018}$

	Lower	0.1180 $_{\pm 0.000.0030}$	0.2996 $_{\pm 0.0013}$	0.4774 $_{\pm 0.0020}$	0.1050 $_{\pm 0.0002}$
	$\Delta^{upper-lower}$	-0.0052 $_{\pm 0.0133}$	-0.0340 $_{\pm 0.0062}$	0.0368 $_{\pm 0.0059}$	0.0026 $_{\pm 0.0024}$

Table S4. Partition coefficients, K, of sodium salts of DNP-amino acids in ATPS indicated (NVal – norvaline, NLeu – norleucine, AA – α-amino-n-octanoic acid)

ATPS	DNP-amino acid Na	K
PEG-PVP	DNP-Ala	0.762 ± 0.005
	DNP-NVal	0.783 ± 0.003
	DNP-NLeu	0.804 ± 0.008
	DNP-AA	0.849 ± 0.009
PEG-PAM	DNP-Ala	1.092 ± 0.007
	DNP-NVal	1.192 ± 0.001
	DNP-NLeu	1.259 ± 0.002
	DNP-AA	1.434 ± 0.001
PEG-Ucon	DNP-Ala	4.283 ± 0.061
	DNP-NVal	5.761 ± 0.035
	DNP-NLeu	7.362 ± 0.102
	DNP-AA	13.00 ± 0.21
PEG-Na_2SO_4	DNP-Ala	3.70 ± 0.070
	DNP-NVal	4.73 ± 0.057
	DNP-NLeu	6.00 ± 0.11
	DNP-AA	10.3 ± 0.19
PEG-Ucon-KCl	DNP-Ala	3.90 ± 0.048
	DNP-NVal	5.52 ± 0.11
	DNP-NLeu	7.29 ± 0.15
	DNP-AA	14.10 ± 0.21
